# Correction of a Serious Error

**Published:** 1904-02

**Authors:** 


### CORRECTION OF A SERIOUS ERROR.

From a number of letters received at this office, it is inferred that a very erroneous opinion exists in the minds of many of the good friends of the International that this journal has been sold to the J. B. Lippincott Company, and, to that extent, has ceased to be a professional journal. We beg to assure those interested in the Journal's welfare that it remains exactly as it has during the years of its existence, in the ownership of the stockholders who originally established it under its present title. Some business arrangements were entered into, to render it, it is hoped, of greater value to the dental profession, but beyond that it will remain, as it always has, free from trade complications, an outspoken vehicle of independent thought and an earnest advocate for that which tends to elevate dentistry wherever it is practised as a profession.

